# Repeatability of ^68^Ga-PSMA-HBED-CC PET/CT–Derived Total Molecular Tumor Volume

**DOI:** 10.2967/jnumed.121.262528

**Published:** 2022-05

**Authors:** Robert Seifert, Patrick Sandach, David Kersting, Wolfgang P. Fendler, Boris Hadaschik, Ken Herrmann, John J. Sunderland, Janet H. Pollard

**Affiliations:** 1 Department of Nuclear Medicine, University of Duisburg–Essen and German Cancer Consortium–University Hospital, Essen, Germany;; 2 West German Cancer Center, Essen, Germany;; 3 Department of Nuclear Medicine, University Hospital Münster, University of Münster, Münster, Germany;; 4 Department of Urology, University Hospital Essen, and German Cancer Consortium–University Hospital, Essen, Germany;; 5 Department of Radiology, University of Iowa Carver College of Medicine, Iowa City, Iowa; and; 6 Department of Radiology, Iowa City Veterans Healthcare Center, Iowa City, Iowa

**Keywords:** PSMA PET, tumor volume, repeatability

## Abstract

Molecular tumor volume (MTV) is a parameter of interest in prostate cancer for assessing total disease burden on prostate-specific membrane antigen (PSMA) PET. Although software segmentation tools can delineate whole-body MTV, a necessary step toward meaningful monitoring of total tumor burden and treatment response through PET is establishing the repeatability of these metrics. The present study assessed the repeatability of total MTV and related metrics for ^68^Ga-PSMA-HBED-CC in prostate cancer. **Methods:** Eighteen patients from a prior repeatability study who underwent 2 test–retest PSMA PET/CT scans within a mean interval of 5 d were reanalyzed. Within-subject coefficient of variation and repeatability coefficients (RCs) were analyzed on a per-lesion and per-patient basis. For the per-lesion analysis, individual lesions were segmented for analysis by a single reader. For the per-patient analysis, subgroups of up to 10 lesions (single reader) and the total tumor volume per patient were segmented (independently by 2 readers). Image parameters were MTV, SUV_max_, SUV_peak_, SUV_mean_, total lesion PSMA, and the related metric PSMA quotient (which integrates lesion volume and PSMA avidity). **Results:** In total, 192 segmentations were analyzed for the per-lesion analysis and 1,662 segmentations for the per-patient analysis (combining the 2 readers and 2 scans). The RC of the MTV of single lesions was 77% (95% CI, 63%–96%). The RC improved to 33% after aggregation of up to 10 manually selected lesions into subgroups assessed per patient (95% CI, 25%–46%). The RC of the semiautomatic MTV_total_ (the sum of all voxels in the whole-body total tumor segmentation per patient) was 35% (95% CI, 25%–50%), the Bland-Altman bias was −6.70 (95% CI, −14.32–0.93). Alternating readers between scans led to a comparable RC of 37% (95% CI, 28%–49%) for MTV_total_, meaning that the metric is robust between scanning sessions and between readers. **Conclusion:** ^68^Ga-PSMA-HBED-CC PET–derived semiautomatic MTV_total_ is repeatable and reader-independent, with a change of ±35% representing a true change in tumor volume. Volumetry of single manually selected lesions has considerably lower repeatability, and volumetry based on subgroups of these lesions, although showing acceptable repeatability, is less systematic. The semiautomatic analysis of MTV_total_ used in this study offers an efficient and robust means of assessing response to therapy.

## Visual Abstract

**Figure:**
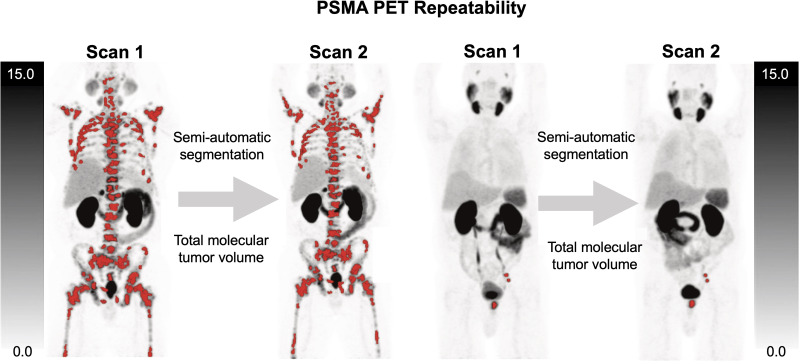

Prostate cancer is a leading cause of death in men (*1*). Especially in advanced prostate cancer, therapy monitoring is challenging. The blood tumor marker prostate-specific antigen is routinely used to monitor disease progression (*2*). However, prostate-specific antigen levels may be influenced by tumor dedifferentiation and androgen deprivation therapy, which raises the need for image-based methods for global tumor assessment (*3*,*4*). For now, bone scanning and CT are the established methods for assessing treatment response in advanced disease (*2*). More recently, prostate-specific membrane antigen (PSMA) imaging with PET has been shown to be superior to conventional imaging for both initial and recurrent cancer staging (*5*,*6*). Therefore, PSMA PET seems to be a promising methodology to quantify the prostate cancer tumor volume over time.

The recently proposed PSMA PET progression criteria, as well as a recently published consensus meeting, recommended consideration of PSMA PET–derived volumetric measurements to detect progressive disease (*7*,*8*). Indeed, several studies have shown that the quantification of the total tumor volume using PSMA PET is feasible and that it is a statistically significant negative predictor for overall survival in patients with advanced prostate cancer (*9*–*12*). Total tumor uptake values analogous to total lesion glycolysis for ^18^F-FDG can also be assessed with PSMA PET.

To date, the repeatability of PSMA PET–derived volumetric and total tumor uptake measurements has not been sufficiently investigated. Previously, Pollard et al. reported ^68^Ga-PSMA-HBED-CC PET repeatability for SUV_max_ in bone and nodal metastases from prostate cancer (*13*). A variety of factors beyond true change in tumor can lead to variability in quantitative PET imaging, including the segmentation methods used. To reliably assess quantitative change between PSMA PET scans, it is necessary to understand the normal variability within the patient, radiotracer, and imaging system. The present study evaluates the repeatability of volumetric and uptake measurements for individual tumors and total tumor volume on test–retest ^68^Ga-PSMA-HBED-CC PET/CT.

## MATERIALS AND METHODS

### Patients and Image Acquisition

Eighteen patients were included in the analysis. The institutional review board approved the study protocol (NCT02952469), and all subjects gave written informed consent. Dataset details were previously reported by Pollard et al. in their study of test–retest repeatability (*13*). Here, the identical dataset was used. Briefly, all patients underwent 2 PSMA PET/CT acquisitions within a mean interval of 5 d (range, 2–14 d). Patient characteristics are shown in Table 1. ^68^Ga-gallium-PSMA-HBED-CC (also known as PSMA-11 and referred to simply as PSMA in the remainder of this paper) was synthesized as previously published (*13*). Either a Biograph mCT (with FlowMotion) or a Biograph TruePoint PET/CT system was used for image acquisition (Siemens Healthineers). The follow-up scan was performed on the same scanner as the initial scan. PET data were acquired using a previously published protocol (PET scan starting 60 min after tracer injection with scan coverage from vertex to mid thigh, 3- to 4-min scan time per bed position) (*13*). A 3-dimensional ordered-subset expectation maximization algorithm was used for image reconstruction (with time-of-flight information in case of the mCT).

**TABLE 1.** Patient Characteristics and MTV_total_ Reported for Each Scan and Reader

			MTV_total_ (mL)			
Patient no.	PSA within ≤90 d (ng/mL)	Gleason score at diagnosis	R1, scan 1	R2, scan 1	R1, scan 2	R2, scan 2
1	0.15	7 (4 + 5)	0	0	0	0
2	4.35	6 (3 + 3)	4.81	5.88	4.81	5.88
3	104.5	9 (4 + 5)	395.7	404.02	399.18	402.22
4	0.14	9 (4 + 5)	59.91	62.59	82.42	66.9
5	0.66	9 (5 + 4)	6.42	6.77	5.18	7.56
6	0.22	9 (5 + 4)	3.78	4.67	3.78	4.67
7	56.3	Presumptive diagnosis	38.89	35.59	41.36	22.49
8	95.5	7 (4 + 3)	206.38	247.85	236.35	221.08
9	276.3	9 (4 + 5)	643.19	741.4	643.19	642.43
10	0.04	Presumptive diagnosis	0	0	0	0
11	0.64	9 (4 + 5)	7.78	8.33	7.78	8.33
12	2.8	Lymph node biopsy	31.49	44.68	30.53	46.24
13	40.1	10 (5 + 5)	464.53	587.13	552.7	515.05
14	19.7	7 (3 + 4)	18.87	22.83	18.87	22.83
15	2.5	Bone biopsy	2.26	1.96	2.26	1.96
16	54.1	9 (5 + 4)	85.89	102.6	92.3	86.56
17	2.5	9 (5 + 4)	21.78	21.81	22.29	21.81
18	2.5	9 (5 + 4)	6.52	6.31	5.53	7.34

PSA = prostate-specific antigen; R1 = reader 1; R2 = reader 2.

### Tumor Analysis per Lesion

For the repeatability analysis of individual lesions, up to 10 metastases (skeletal or nodal) or primary tumor lesions were segmented in both the first and second scans by a single reader using a manual segmentation with a 50% isocontour. A single-reader model was chosen for the single-lesion and the subgroup analysis portions of the study. Because the same small number of lesions needed to be selected on each PET scan, the single-reader approach minimized variability introduced by interrater differences in lesion selection and segmentation. Lesions were identified as nonphysiologic sites of uptake with an SUV exceeding the regional background activity. Lesions were selected at random from the regions segmented by the whole-body molecular tumor volume (MTV) analysis, described in detail in the section on total tumor analysis. For each lesion, SUV_max_, SUV_peak_, SUV_mean_, lesion MTV (MTV_lesion_), total lesion PSMA (PSMA-TL_lesion_), and total lesion quotient (PSMA-TLQ_lesion_) were measured. MTV_lesion_ was determined by the sum of the voxels (Eq. 1) within a threshold 50% isocontour of the local SUV_max_. PSMA-TL_lesion_ and PSMA-TLQ_lesion_ were calculated as in Equations 2 and 3.

$${\text{MTV}}_{\text{lesion}}=\sum_{i=0}^{\text{total}}\left({\text{voxel}}_{i}\right)$$ Eq. 1

$$\text{PSMA}-{\text{TL}}_{\text{lesion}}={\text{MTV}}_{\text{lesion}}\;\times {\text{lesion}\;\text{SUV}}_{\text{mean}}$$ Eq. 2

$$\text{PSMA}-{\text{TLQ}}_{\text{lesion}}=\frac{{\text{MTV}}_{\text{lesion}}}{{\text{lesion}\;\text{SUV}}_{\text{mean}}}$$ Eq. 3

### Tumor Subgroup Analysis per Patient

One reader manually selected at random a group of up to 10 lesions per patient from the regions segmented by the whole-body MTV analysis technique (described in the next section). In patients with a large number of metastatic lesions, lesions were selected randomly to reflect a broad distribution of anatomic regions. The lesions in this subgroup were individually manually segmented and were assessed as an aggregate. The mean of SUV_max_ and SUV_mean_ (subgroup mean SUV_max_ and subgroup mean SUV_mean_, respectively) were calculated. The sum and mean of MTV_lesion_, the sum of PSMA-TL_lesion_, and the sum of PSMA-TLQ_lesion_ (MTV_subgroup_, subgroup MTV_mean_, PSMA-TL_subgroup,_ and PSMA-TLQ_subgroup_, respectively) were calculated as in Equations 4–7, where *n* is the number of lesions within the subgroup and *i* is the ordinal number of the lesion. PSMA-TL_lesion_ is analogous to total lesion glycolysis for ^18^F-FDG and, when calculated for aggregate tumors, the individual PSMA-TL_lesion_ values are summed.

$${\text{MTV}}_{\text{subgroup}}=\sum_{i=1}^{n}\left({\text{MTV}}_{\text{lesion}\;i}\right)$$ Eq. 4

$${\text{Subgroup}\;\text{MTV}}_{\text{mean}}=\frac{\sum_{i=1}^{n}({\text{MTV}}_{\text{lesion}\;i\;})}{n}$$ Eq. 5

$$\text{PSMA}-{\text{TL}}_{\text{subgroup}}=\sum_{i=1}^{n}\left(\text{PSMA}-{\text{TL}}_{\text{lesion}\;i}\right)\;\;$$ Eq. 6

$$\text{PSMA}-{\text{TLQ}}_{\text{subgroup}}=\sum_{i=1}^{n}\;(\text{PSMA}-{\text{TLQ}}_{\text{lesion}\;i})\;$$ Eq. 7

### Total Tumor Analysis per Patient

**FIGURE 1.**
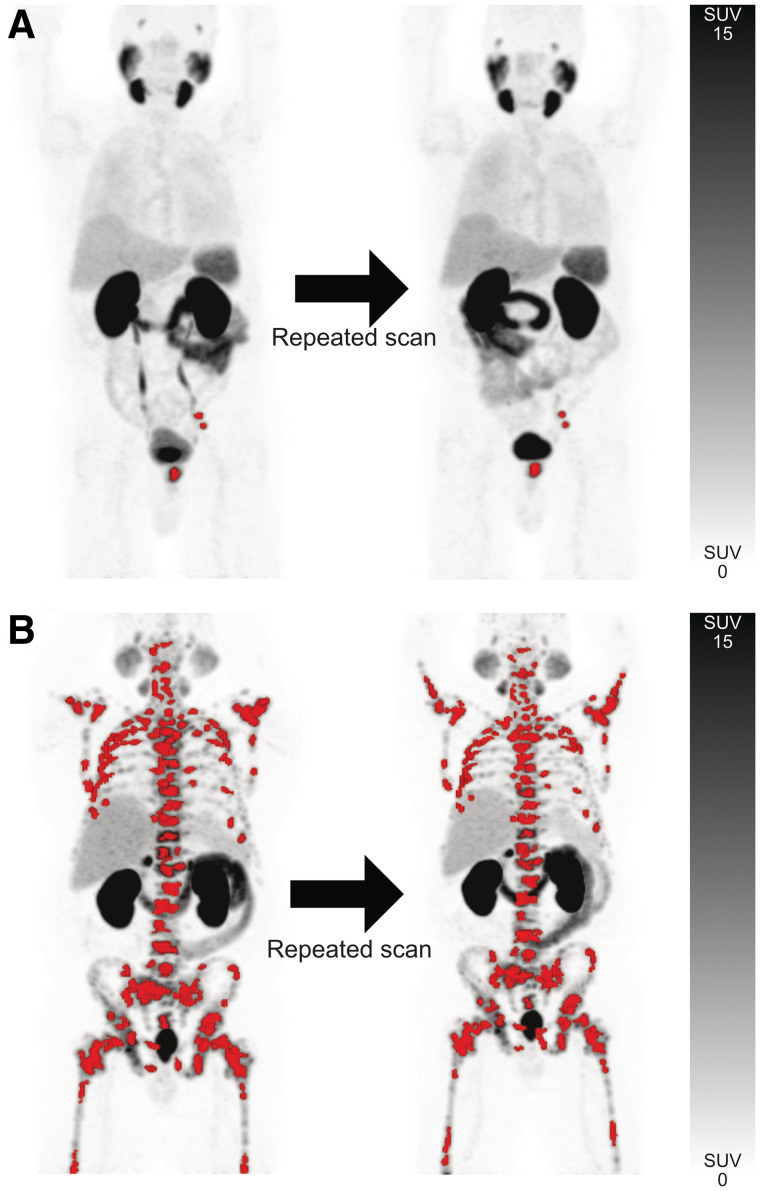
Semiautomatic total tumor segmentations with red overlay designating sites of segmented lesions in scans 1 and 2 for patient with disease limited to prostate and left pelvic lymph nodes (A) and patient with extensive skeletal metastases (B). Interval between scans was 2 d for both patients.

**FIGURE 2.**
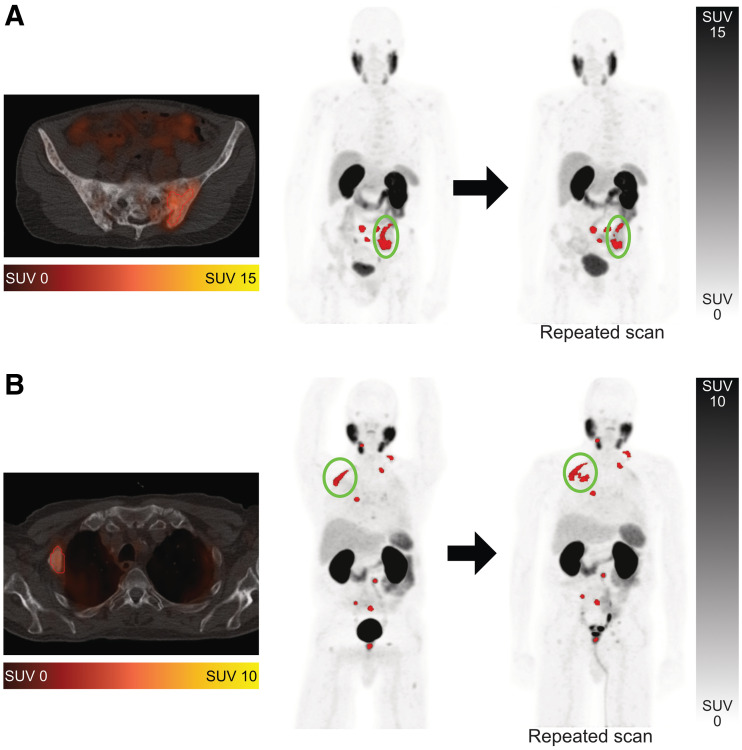
Examples of segmentation challenges on ^68^Ga-PSMA-HBED-CC PET/CT. Segmented tumor metastases are shown in red. (A) Metastasis in os ilium was segmented as single lesion on first scan but as 3 separate lesions in second scan (encircled). (B) Metastasis in rib was segmented accurately on first scan but inaccurately on second scan, with isocontour including portion of lung (encircled). Error was resolved manually.

For the total tumor analysis, all lesions were segmented using a semiautomatic approach as previously published (*9*). The investigational MICIIS research software prototype was used for the single lesion and total tumor analyses (previously named MI Whole-Body Analysis Suite; Siemens Healthineers). Briefly, all voxels with an SUV_peak_ exceeding the following liver-specific threshold were selected as candidate foci:

$${\text{SUV}}_{\text{peak}}\text{threshold}\;\geq \frac{4.3}{{\text{liver}\;\text{SUV}}_{\text{mean}}}\;\times ({\text{liver}\;\text{SUV}}_{\text{mean}}+{\text{liver}\;\text{SUV}}_{\text{SD}}),$$ Eq. 8

where the liver-specific threshold was calculated as previously described and SUV_SD_ is the SD of the SUV distribution in the liver volume of interest (*9*,*10*). The threshold described in Equation 8 adjusts for the tumor sink effect, which has a tendency to lower liver uptake; the first part of the formula is a corrective coefficient for the SUV reduction due to the sink effect, and the second part is the calculation for the uncorrected liver threshold. Individual-lesion segmention was based on a threshold 50% isocontour of the local SUV_max_. In analogy to the European Association of Nuclear Medicine recommendations for ^18^F-FDG PET imaging, a threshold 50% isocontour-based approach was chosen for this study (*14*). Segmentation errors such as inclusion of sites of normal physiologic uptake or exclusion of tumor lesions were adjusted manually. There were no adjustments of segmented tumor contours in addition to inclusion or exclusion of lesions. Example whole-body tumor segmentations and segmentation errors are shown in Figures 1 and 2. Two readers with PET experience independently delineated all tumor lesions, and their delineated PET data were analyzed separately.

The sum of all voxels in the whole-body total tumor segmentation per patient was designated MTV_total_. The mean of the volume of the individual segmented volumes comprising the MTV_total_ was calculated as in Equation 5 and was designated total MTV_mean_. Likewise, the mean of SUV_max_ and SUV_mean_ of these component volumes was designated total mean SUV_max_ and total mean SUV_mean_, respectively. The PSMA-TL_lesion_ and PSMA-TLQ_lesion_ values for the component volumes were summed as in Equations 6 and 7 and were designated PSMA-TL_total_ and PSMA-TLQ_total_, respectively.

### Statistical Analysis

Statistical methods for the sample size of the original dataset used in this analysis were reported by Pollard et al. (*13*). The Pearson correlation coefficient was used for descriptive statistics. Bland–Altman plots were created for absolute (rather than relative percent) differences in MTV_total_ and mean SUV_max_ (*15*). Correlation in MTV_total_ between readers for the same scan and between scans for the same reader was evaluated with intraclass correlation coefficients. The repeatability assessment using a relative comparison approach was done as described by Obuchowski (*16*). The within-subject coefficient of variation (wCV) is given by

$$\text{wCV}=\;\sqrt{\frac{\sum_{i=1}^{n}\frac{{\left({\text{scan}\;\text{A}}_{i}-{\text{scan}\;\text{B}}_{i}\right)}^{2}}{2\times {\left(\frac{1}{2}\left({\text{scan}\;\text{A}}_{i}+{\text{scan}\;\text{B}}_{i}\right)\right)}^{2}}}{n}}$$ Eq. 9

where *n* is the number of subjects and scans A and B are the quantitative PET measurements from the first and second PET scans, respectively. The repeatability coefficient (RC) is given by

$$\text{RC}=1.96\times \text{wCV}\times \sqrt{2}$$ Eq. 10

The CIs for wCV and RC were determined by bootstrapping with 1,000 replicates. RC variability in relation to lesion SUV_max_ was evaluated by an exploratory approach for subsets of lesions in multiple steps. For each step, all lesions that had an SUV_max_ below an arbitrarily defined SUV_max_ threshold were included. A distinct threshold was used for each step; the lowest SUV_max_ threshold was 1, and the increment was 5. Statistical analyses were performed using R, version 3.5.2 (The R Foundation, https://www.r-project.org/) and Microsoft Excel 2016, version 16.0.5110.1000. Statistical analysis was done by David Kersting and Robert Seifert.

## RESULTS

### Test–Retest Scan Parameters

As previously published for the same cohort, the median interval between scans 1 and 2 was 5 d (range, 2–14 d). No statistically significant difference between scans 1 and 2 was observed regarding injected dose (mean, 133.1 vs. 133.1 MBq; *P* = 1.0) or image delay (mean, 60. 6 vs. 60.7 min; *P* = 0.9). Patient characteristics are shown in Table 1.

### Repeatability of Manually Segmented Individual Lesions

**FIGURE 3.**
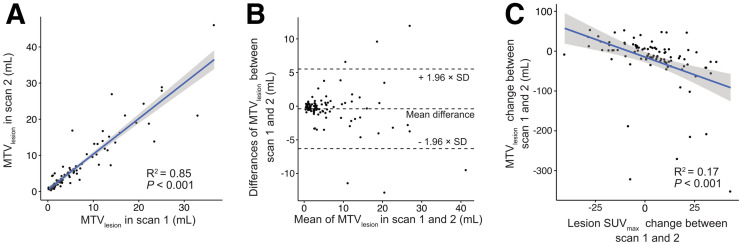
Analysis of individual manually segmented ^68^Ga-PSMA-HBED-CC–avid lesions. Linear regression and Bland–Altman plots (A and B) of MTV_lesion_ show correlation between scans. (C) Association is noted between MTV_lesion_ and SUV_max_ changes between scans 1 and 2.

For the per-lesion analysis, 96 metastases from 18 patients were manually delineated by a single reader, resulting in a total number of 192 segmentations from the 2 scans. Segmented lesions were regarded as independent observations. The RCs of MTV_lesion_ and related metrics are shown in Table 2. Linear regression and Bland–Altman scatterplots for MTV_lesion_ on scans 1 and 2 showed a relatively strong correlation (*P* < 0.001, *R*^2^ = 0.85) and no significant bias based on visual analysis (Figs. 3A and 3B). However, MTV_lesion_ demonstrated poor repeatability, with an RC of 76.9% (95% CI, 62.9%–95.9%), and similarly poor repeatability when accounting for differences in lesion volume, with an RC of 64.7% (range, 49.3%–91.6%), for lesions 5 cm^3^ or larger and 83.9% (range, 65.5%–110.7%) for lesions smaller than 5 cm^3^. The Bland–Altman bias of MTV_lesion_ was −0.39 (95% CI, −1.00 to 0.22) for all lesions. The deviation in MTV_lesion_ between scans 1 and 2 correlated to a statistically significantly extent with the deviation in SUV_max_ between scans 1 and 2 (*P* < 0.001, *R*^2^ = 0.17) (Fig. 3C).

**TABLE 2.** Repeatability of Manually Segmented Individual Lesions (MTV_lesion_)

Metric	wCV (%)	RC (%)	95% CI of RC (%)
MTV_lesion_	27.7	76.9	62.9–95.9
PSMA-TL_lesion_	23.3	64.7	53.4–80.67
PSMA-TLQ_lesion_	34.5	95.7	81.5–114.5
Lesion SUV_max_	12.4	34.4	29.6–41.2
Lesion SUV_peak_	9.9	27.3	23.3–32.8
Lesion SUV_mean_	11.8	32.7	27.5–40.2

### Repeatability of Manually Segmented Subgroup of Lesions per Patient

Given the poor repeatability of MTV_lesion_, a larger subgroup of manually selected and segmented lesions was evaluated for repeatability per patient. Inclusion of multiple lesions for assessment as a subgroup allows for mitigation of individual lesion variability by averaging positive and negative variation across a larger number of lesions. The repeatability of MTV_subgroup_ and related metrics is presented in Table 3. MTV_subgroup_ demonstrated improved repeatability, with an RC of 33.1% (95% CI, 24.2%–46.2%), compared with MTV_lesion_ and showed a repeatability comparable to that of the semiautomatic whole-body approach of MTV_total_. The Bland–Altman bias of MTV_subgroup_ was −2.32 (95% CI, −5.81 to 1.17). Supplemental Table 1 shows the association of RC, with SUV_max_ RC decreasing with increasing minimum SUV_max_ of segmented lesions (supplemental materials are available at http://jnm.snmjournals.org). This finding indicates that the repeatability was better when lesions with a low SUV_max_ were discarded from the manually segmented subgroup of lesions.

**TABLE 3.** Repeatability of Manually Selected Lesion Subgroup per Patient (MTV_subgroup_)

Metric	wCV (%)	RC (%)	95% CI of RC
MTV_subgroup_	12.0	33.1	24.2–46.2
Subgroup MTV_mean_	12.0	33.1	24.8–47.7
PSMA-TL_subgroup_	7.4	20.6	16.0–26.9
PSMA-TLQ_subgroup_	18.4	51.0	36.5–78.0
Subgroup mean SUV_max_	12.3	34.0	20.0–59.4
Subgroup mean SUV_peak_	6.6	18.3	13.3–24.5
Subgroup mean SUV_mean_	9.1	25.2	17.5–35.7

### Repeatability of Semiautomatic Segmentation of Total Tumor Volume per Patient

**FIGURE 4.**
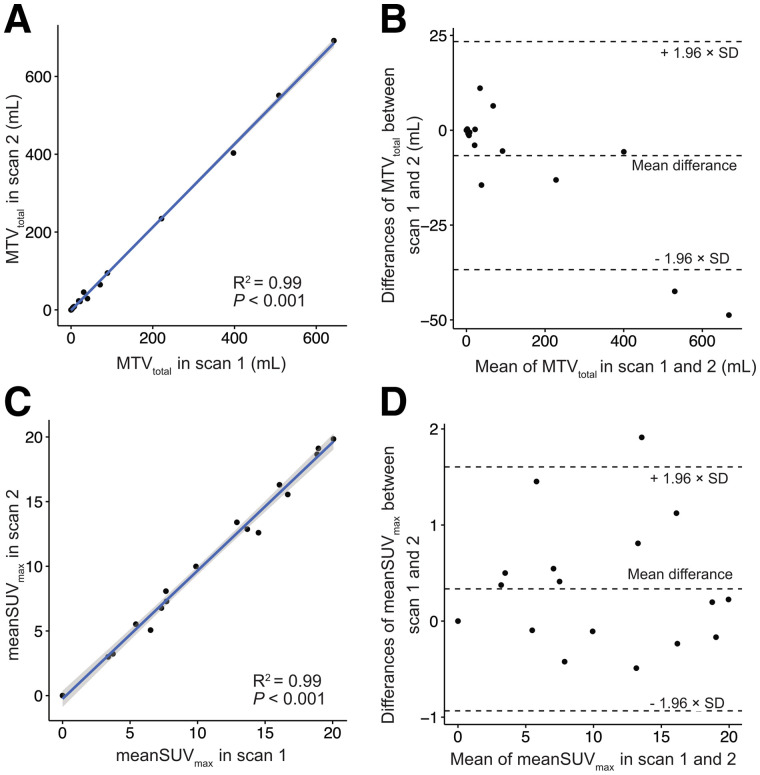
Analysis of semiautomatic whole-body segmentation of ^68^Ga-PSMA-HBED-CC–avid lesions. Linear regression (A and C) and Bland–Altman plots (B and D) of MTV_total_ and mean SUV_max_ show excellent correlation between scans and suggest no association between total tumor volume or lesion intensity and test–retest differences. Results for readers 1 and 2 were averaged for purposes of these graphs. (A and C) MTV_total_ and mean SUV_max_ for scan 1 are plotted separately against same metric for scan 2. (B and D) Mean of MTV_total_ or mean SUV_max_ between scans 1 and 2 was plotted against absolute difference in metric between 2 scans.

**FIGURE 5.**
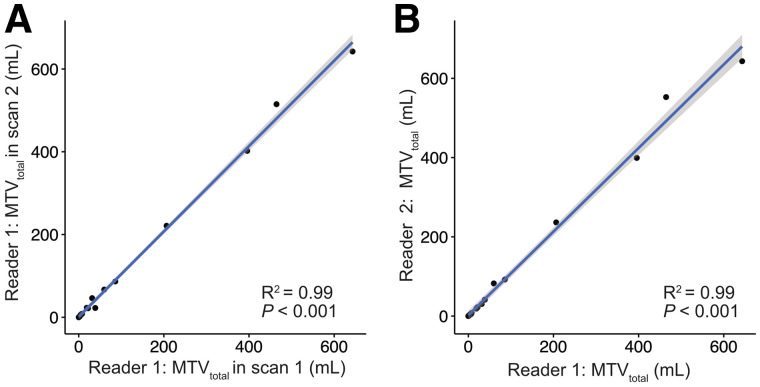
Graphical analysis of intra- and interreader agreement in reporting MTV_total_, showing high correlation in measures between scans 1 and 2 for same reader (reader 1) (A) and showing high correlation in measures between 2 independent readers for same scan (scan 1) (B).

**FIGURE 6.**
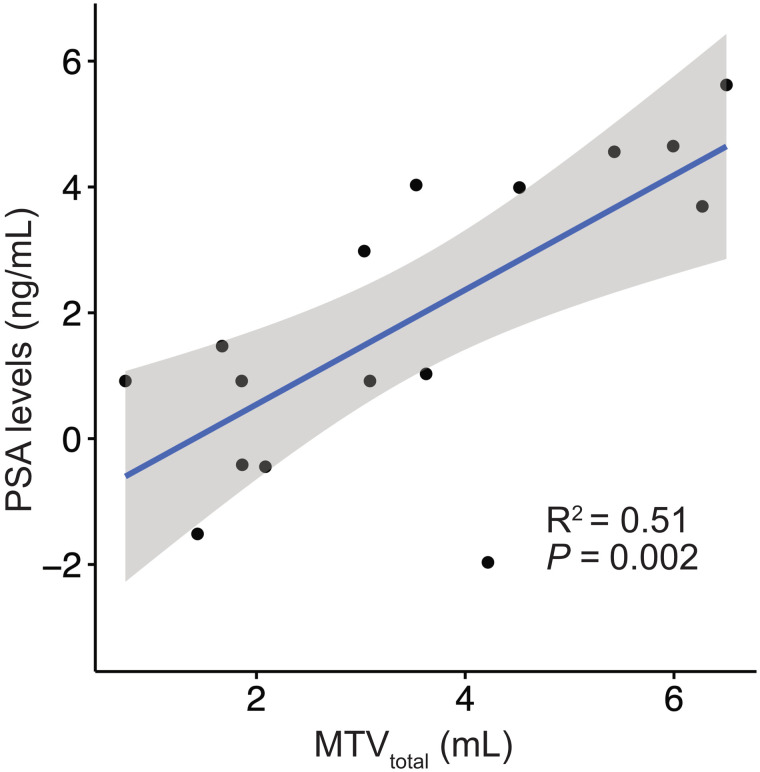
Graphical analysis of prostate-specific antigen vs. MTV_total_, with log–log plot showing moderate correlation.

In total, 1,662 segmentations were performed for the per-patient analysis, including segmentations for the 2 readers and 2 scans. The MTV_total_ for each reader for scans 1 and 2 is presented in Table 1. The RCs of the whole-body MTV_total_ and related metrics are shown separately for both readers in Table 4. The RC of MTV_total_ was 35.0% (95% CI, 24.9%–49.7%) in mean; the RCs for each reader were 37% and 33%. Linear regression and Bland–Altman scatterplots for MTV_total_ and mean SUV_max_ for scans 1 and 2 showed a strong correlation (*P* < 0.001, *R*^2^ = 0.99) and no significant bias based on visual analysis (Fig. 4). The corresponding Bland–Altman bias of MTV_total_ was −6.70 (95% CI, −14.32 to 0.93). The RC of MTV_total_ and related metrics remained robust even when readers were hypothetically exchanged between scan timepoints with an RC of MTV_total_ of 37.3% (95% CI, 27.9%–49.3%) (Table 5). A high correlation of MTV_total_ between scans for the same reader (intraclass correlation coefficient, 0.998; *P* < 0.001) and between readers for the same scan (intraclass correlation coefficient, 0.993; *P* < 0.001) was noted (Fig. 5). MTV_total_ showed a moderate correlation with prostate-specific antigen values (*P* < 0.002, *R*^2^ = 0.53) (Fig. 6). Other metrics using the semiautomatic technique, such as total mean SUV_max_, total mean SUV_mean_, and PSMA-TL_total_, also showed improved repeatability as compared with individual lesion segmentation, with RC ranging from 23.6% to 28.4%.

**TABLE 4.** Repeatability of Semiautomatic MTV_total_ per Patient

Metric	R1 wCV (%)	R2 wCV (%)	Mean wCV (%)	R1 RC (%)	R2 RC (%)	Mean RC (%)	95% CI of mean RC
MTV_total_	13.4	11.9	12.7	37.0	33.0	35.0	24.9–49.7
Total MTV_mean_	13.4	11.9	12.7	37.1	33.0	35.0	25.0–48.8
PSMA-TL_total_	8.4	12.1	10.3	23.3	33.5	28.4	20.7–41.9
PSMA-TLQ_total_	19.4	17.3	18.4	53.9	48.0	50.9	32.7–84.7
Total mean SUV_max_	8.4	8.6	8.5	23.3	23.9	23.6	17.0–32.4
Total mean SUV_mean_	8.1	8.0	8.1	22.6	22.2	22.4	16.4–30.7

R1 = reader 1; R2 = reader 2.

**TABLE 5.** Repeatability of MTV_total_ with Different Readers Between Scans

Metric	R1, R2 RC (%)	R2, R1 RC (%)	Mean RC (%)	95% CI of mean RC
MTV_total_	29.9	44.7	37.3	27.9–49.3
Total MTV_mean_	29.9	44.7	37.3	29.9–44.7
PSMA-TL_total_	24.9	37.2	31.0	24.5–39.5
PSMA-TLQ_total_	52.5	58.4	55.5	38.1–83.6
Total mean SUV_max_	28.3	20.7	24.5	17.5–33.5
Total mean SUV_mean_	27.4	18.7	23.1	17.2–31.1

R1, R2 = first scan read by reader 1, second scan read by reader 2; R2, R1 = first scan read by reader 2, second scan read by reader 1.

## DISCUSSION

PSMA PET is now widely used to monitor patients with prostate cancer (*5*,*6*,*17*,*18*). Especially for recurrent prostate cancer, PSMA PET has demonstrated high sensitivity and specificity for localizing prostate cancer cells in the body (*5*,*6*). Growing evidence suggests that PSMA PET is also a useful clinical tool in patients with more advanced prostate cancer (*19*–*21*).

Measures of total tumor volume and total uptake on PSMA PET have been described, but its use in prostate cancer monitoring remains under debate (*9*,*10*). Analogous to the TNM system, a molecular imaging TNM (miTNM) system has been proposed, which scores extent of disease with regard to local tumor, regional lymph node metastases, bone metastases, and other distant metastases. (*22*). Given the distinct biologic aggressiveness and survival implications of various metastatic sites, the TNM-based system will likely remain an important prognostic tool in prostate cancer (*23*). However, because progressive disease is not always accompanied only by the occurrence of new metastases but also can include enlargement of existing metastases, the best assessment tools would therefore encompass assessment of both anatomic and total tumor volume, enabling consideration of both global disease status and aggressiveness of the involved sites. Studies have shown the prognostic value of PET volumetry and measures of total uptake. PSMA MTV_total_ of high volume disease is a statistically significant poor prognostic factor for overall survival, and PSMA uptake of all metastases (SUV_mean_ per patient) improves prognostication of overall survival in patients treated with ^177^Lu-lutetium-PSMA-617 therapy (*12*,*24*–*27*).

The recently proposed PSMA PET progression criteria recommend assuming progressive prostate cancer in the setting of a 30% tumor volume increase (*8*). However, this threshold was chosen arbitrarily in the absence of volume-based PSMA repeatability data. Also, there are currently no consensus recommendations for PSMA PET segmentation algorithms among the various approaches that have been proposed for quantifying the PSMA tumor volume (*9*,*10*). A necessary step toward use of PSMA PET for reliable monitoring of disease is development of reliable and efficient methods for measuring total disease burden and determination of their repeatability.

Our analysis of repeatability evaluated PET volumetric and uptake measures using 3 different approaches to segmentation: manual segmentation of individual tumors, manual selection of a subgroup of tumors per patient, and semiautomatic segmentation of total tumor burden per patient. The repeatability of individual tumor volumes (MTV_lesion_) was poor (RC, 77%). An explanation may be that the reported wCV for SUV_max_ (12%–14%, Pollard et al. (*13*)), combined with a volumetric measurement in which a small change in radius from the 50% SUV_max_ threshold results in a large change in volume, predictably results in large variability. Therefore, monitoring disease on the basis of individual manually segmented tumors does not appear to be a reliable marker for treatment response. The RC for subgroup MTV_mean_ and total MTV_mean_ was 33% and 35%, respectively, which is similar to that reported in the literature for ^18^F-FDG for other cancers (*28*,*29*). An MTV based on a larger sample of tumors or total tumor volume rather than individual tumors appears to be more reliable, likely because the noise-sensitive SUV_max_-based thresholds and resulting volume differences have both plus and minus biases across all lesions, resulting in a tendency to cancel out. Although robust, the method based on selection of a subgroup of tumors would be time-consuming in clinical practice and prone to bias in lesion selection. Because of the limited data available, no clear recommendation for a minimum number of lesions for the subgroup of lesions can be made; moreover, the repeatability of quantified volume is likely influenced by the characteristics of the chosen lesions (e.g., lesion size and tissue type). MTV_total_ remains robust even when alternating readers between baseline and follow-up scans, suggesting that this method would hold up in clinical practice when scans are not always read by the same person. Therefore, the standardized semiautomatic segmentation method for MTV_total_ proposed by Seifert et al., which worked well in this study, may be a solution (*9*). Future investigation should focus on the fully automatic analysis of PSMA PET scans in analogy to ^18^F-FDG PET approaches (*30*). MTV_total_ showed a moderate correlation with prostate-specific antigen, suggesting that further assessment of this metric for use as a surrogate biomarker for disease status is warranted.

Besides volumetry, we evaluated SUV measures, which showed repeatability similar to that reported by Pollard et al. (*13*). We also evaluated PSMA-TL and PSMA-TLQ, metrics that integrate tumor volume and uptake analogous to total lesion glycolysis for ^18^F-FDG. These metrics showed poor repeatability in individual lesions, but improved repeatability for the subgroup of tumors and total tumor burden, and thus warrant further investigation (*12*). Interestingly, PMA-TLQ had greater variability than PSMA-TL. This might be partly explained by the fact that the tumor volume is normalized with the relatively stable (i.e., high-repeatability) SUV_mean_. Thereby, changes in the tumor volume have a larger influence on the resulting composite metric.

The present study had some limitations. The fact that patient number was relatively small might influence the translatability to a larger patient population. The results might not be directly translatable to other PSMA ligands, especially to those that are conjugates with nongallium radioisotopes. The segmentation technique may cause difficulties when single lesions are segmented separately in follow-up scans or when confluent lesions occur (Fig. 2). However, manual user-dependent adjustments can eliminate those artifacts. Finally, the test–retest dataset was performed under carefully controlled conditions (e.g., ensuring the same scanner for scans 1 and 2, minimizing variation in uptake time and dose), which do not reflect the potential variations encountered in the real-world clinic setting.

## CONCLUSION

^68^Ga-PSMA-HBED-CC PET–derived MTV_total_ with semiautomatic whole-body segmentation is highly repeatable and suitable for monitoring disease in advanced prostate cancer. Other methods evaluated in this study, such as single-lesion volumes and subgroup of lesions per patient, are limited by inferior repeatability (MTV_lesion_) or labor intensiveness (MTV_subgroup_). MTV_total_ therefore presents an efficient and robust means of monitoring disease longitudinally. A change of greater than 35% in the magnitude of MTV_total_ can be viewed as a real change in tumor status progression or response to therapy.

## DISCLOSURE

Janet H. Pollard has been an investigator for Progenics (Advanced Accelerator Applications) and Endocyte (Novartis) and has received compensation for work done for KEOSYS/Exini. Boris Hadaschik reports a consulting or advisory role at ABX, Astellas Pharma, Bayer, Bristol-Myers Squibb, Janssen, and Lightpoint Medical, Inc.; research funding from Astellas Pharma, Bristol-Myers Squibb, German Cancer Aid, and the German Research Foundation; and travel accommodations and expenses from Astellas Pharma, AstraZeneca, and Janssen. Wolfgang Fendler was a consultant for BTG and received fees from RadioMedix, Bayer, and Parexel outside the submitted work. No other potential conflict of interest relevant to this article was reported.

## Notes

KEY POINTS **QUESTION:** What is the estimated test–retest repeatability of whole-body MTV_total_ for ^68^Ga-PSMA-HBED-CC PET/CT in patients with metastatic prostate cancer? **PERTINENT FINDINGS:** This study evaluated the test–retest repeatability of semiautomatic segmentation of whole-body MTV_total_, showing a wCV of 12.7% and an RC of ±35%. The repeatability of manually segmented individual tumors (MTV_lesion_) was poor, whereas the repeatability of a manually selected subgroup of tumors per patient (MTV_subgroup_) was robust but limited by labor intensiveness. **IMPLICATIONS FOR PATIENT CARE:** Understanding test–retest repeatability for metrics of metastatic disease burden is important for the development of ^68^Ga PSMA HBED-CC PET/CT as a quantitative imaging biomarker. This study suggests that semiautomatically segmented whole-body MTV_total_ is efficient and robust for monitoring disease status.
